# Subacute Thyroiditis: Clinical Presentation and Long Term Outcome

**DOI:** 10.1155/2014/794943

**Published:** 2014-04-03

**Authors:** Assim A. Alfadda, Reem M. Sallam, Ghadi E. Elawad, Hisham AlDhukair, Mossaed M. Alyahya

**Affiliations:** ^1^Department of Medicine, College of Medicine, King Saud University, P.O. Box 2925 (38), Riyadh 11461, Saudi Arabia; ^2^Clinical Chemistry Unit, Department of Pathology, College of Medicine, King Saud University, P.O. Box 2925 (30), Riyadh 11461, Saudi Arabia; ^3^Department of Medical Biochemistry, Faculty of Medicine, Ain Shams University, Abbassia, Cairo 11566, Egypt; ^4^National Neuroscience Institute, King Fahad Medical City, Riyadh 11525, Saudi Arabia; ^5^Neurology Department, King Faisal Specialist Hospital & Research Center, Riyadh 11211, Saudi Arabia

## Abstract

Few studies have been reported from the Kingdom of Saudi Arabia (SA) to describe the clinical presentation and long term outcomes of subacute thyroiditis (SAT). Our aim was to review the demographic, anthropometric, clinical presentation, laboratory results, treatment, and disease outcome in Riyadh region and to compare those with results from different regions of the Kingdom and different parts of the world. We reviewed the medical files of patients who underwent thyroid uptake scan during an 8-year period in King Khalid University Hospital. Only 25 patients had confirmed diagnosis of thyroiditis. Age and gender distribution were similar to other studies. Most patients presented with palpitation, goiter, and weight change. Elevated thyroid hormones, suppressed thyroid-stimulating hormone, and elevated ESR were reported. Among those, 7 cases of SAT were recorded. **β**-Blockers were prescribed to 57% and nonsteroidal anti-inflammatory drugs to 29% of SAT. Long follow-up demonstrated that 85.7% of SAT cases recovered, while 14.3% developed permanent hypothyroidism. In conclusion, SAT is uncommon in the central region of SA. Compared to the western region, corticosteroid is not commonly prescribed, and permanent hypothyroidism is not uncommon. A nation-wide epidemiological study to explain these interprovincial differences is warranted.

## 1. Introduction

Thyroiditis, which literally means thyroid inflammation, is a term that includes different conditions with some sort of controversy in nomenclature even among thyroid disorders expert endocrinologists. Thyroiditis disorders can be classified in several ways, based on different etiology—whether known or suspected—different pathology, or different clinical presentation. They include painful conditions as subacute thyroiditis (SAT) and suppurative thyroiditis and painless conditions in which the primary manifestations are thyroid dysfunction or enlargement (goiter) in the absence of clinically evident manifestations of acute inflammation. Examples of the second group are painless thyroiditis and Riedel's (fibrous) thyroiditis [1].

SAT, also called subacute granulomatous or de Quervain thyroiditis, is an uncommon condition, yet is considered the most common cause of painful thyroiditis. The disease is thought to have a viral origin, with possible pathogens including mumps virus, hepatitis B and C viruses, cytomegalovirus, enterovirus, and coxsackie viruses A and B [2]. However, the exact etiology of SAT is unknown. Studying the natural history and clinical outcome of the disease in different ethnic backgrounds has a potential significance since genetic factors that might affect individuals' susceptibility to possible viral pathogens were reported to play a role in the pathogenesis of the disease [3]. Clinically, the condition is associated with severe pain that is usually localized to the anterior aspect of the neck and may radiate up to the jaw or ear. In addition, tenderness of the thyroid gland upon palpation and small diffuse goiter are frequently present. Common initial clinical features and laboratory investigation results include low-grade fever, fatigue, mild thyrotoxic manifestations, suppressed thyroid stimulating hormone (TSH), poor or no thyroid uptake, and elevated erythrocyte sedimentation rate (ESR). Within few weeks and after the depletion of the preformed thyroid hormone, about 30% of patients will undergo a hypothyroid phase. The latter might last up to several months to be followed by an euthyroid phase. Clinical picture and laboratory results are the bases for the diagnosis, while histopathology and cytological diagnosis are rarely required [4].

The uncommon incidence of the disease is reported in several parts of the world. In a reasonably comprehensive study that was published in 2003 from Olmsted County in Minnesota, USA, the incidence rate for the disease was reported to be 3.6 cases per 100,000/year in the most recent years of the study, with the incidence rate for women exceeding that for men. The peak incidence was in the fifth decade of life for both sexes [5]. From Italy, a study was recently published that included patients retrospectively evaluated at an Endocrine and Metabolic Unit in a single institution during 2 years. The authors have demonstrated that 0.3% of those patients were diagnosed as SAT, also with a differential gender preference toward female (female to male ratio of ~6 : 1) [6].

There are few available studies that focus on the demographic distribution and clinical outcome of SAT in the Kingdom of Saudi Arabia. About a decade ago, Qari and Maimani have published their work studying the disease outcome in the western province of the Kingdom, an area that is located along the Red Sea shore and as such represents a different environment and lifestyle nature than those prevalent in the central region of the country [7]. The study has followed up 23 patients with SAT for 2-year duration and commented on the clinical presentation and outcome of the disease in this area of the country.

The current study was designed with the objectives to report the clinical presentation, laboratory investigations, therapeutic approach, and clinical outcome of SAT in the region of the capital city: Riyadh (central province of the Kingdom of Saudi Arabia) and to compare these data with the results previously published from the western province of the country and to those reported from different parts of the world. A long follow-up for the patients' clinical outcome was an important priority in the design of this study to monitor potential long term thyroid dysfunction. To fulfill these objectives, we designed a retrospective review study in King Khalid University Hospital (KKUH) of King Saud University (KSU) located in Riyadh, where we retrieved relevant medical information from the Medical Record Department of KKUH looking at medical records of all patients who underwent a thyroid uptake scan during an eight-year duration. Initially, we have analyzed the data of all thyroiditis cases retrieved in our search; then we focused on the confirmed cases of SAT.

## 2. Materials and Methods

This is a retrospective review study that was performed in the Obesity Research Center, KKUH, KSU, Riyadh. After obtaining the institutional Ethical Committee approval, we retrieved relevant medical information from the Medical Records Department of the hospital looking at all patients who underwent a thyroid uptake scan between January 1, 2004, and December 31, 2011. The aim was that to study various forms of thyroiditis and specifically SAT in the region of Riyadh.

Based on the retrieved medical information, subjects were included if they manifest low normal, reduced, or absent 99m technetium pertechnetate (Tc-99m) thyroid uptake with no detected visible nodules on thyroid scan. We have chosen the thyroid uptake as an initial means for medical record retrieval because it is the investigation tool most frequently requested for suspected cases of thyroiditis in our center. For those patients, the diagnosis of different types of thyroiditis was recorded from their medical files, and based on the classification published by Pearce and colleagues, as follows [1]. Hashimoto's thyroiditis was diagnosed by the presence of high persistent titer of antithyroid antibodies as antimicrosomal (AMA) or antithyroglobulin (ATA) antibodies and the characteristic pathological findings of lymphocytic infiltration, germinal centers, or fibrosis, when available. Painless postpartum thyroiditis was diagnosed in women of childbearing age, who showed low thyroid uptake, high titer of antithyroid autoantibodies, and characteristic lymphocytic infiltration when pathological analysis was recorded, while thyroid function might show variable findings. Painless sporadic thyroiditis diagnostic criteria are very similar to the painless postpartum thyroiditis and can manifest in male as well. Drug-induced thyroiditis, as the name implies, occurs secondary to certain drugs as amiodarone, lithium, interferon *α*, or interleukin-2. Painful thyroiditis includes SAT, suppurative thyroiditis, radiation-induced thyroiditis, and trauma- or palpation-induced thyroiditis. SAT diagnostic criteria include absent or low thyroid uptake in the thyroid uptake scan and the presence of transient thyrotoxicosis at time of presentation in the absence of both excessive iodine intake history and history of medication that could explain the transient thyrotoxicosis (e.g., lithium, amiodarone, and various cytokines). Hypothyroidism or even euthyroidism might be detected depending on the natural history of the disease and the time of presentation. In addition to abnormal thyroid uptake and the thyroid function test (TFT) results, painful SAT diagnosis, as the name implies, was based on fulfilling two other criteria, namely, neck pain and/or tenderness and elevated ESR. Suppurative thyroiditis is an infectious disease with potential abscess formation. While the ESR is elevated, together with other manifestation of acute infection in suppurative thyroiditis, both the thyroid uptake and the TFT can be normal.

Information regarding the subjects demographic criteria; age at presentation, sex, and socioeconomic state; family and previous medical history; clinical picture at presentation and results of laboratory investigations including ESR, TFT, and antithyroid antibody measurements were all recorded. The reported TFT including TSH, FT4, and FT3 were performed in KKUH using commercially available kits by Roche Elecsys modular analytics Cobas e411 utilizing electrochemiluminescence immunoassay (Roche Diagnostics, Mannheim, Germany). Specific thyroid-related investigations in addition to the thyroid uptake scan, as thyroid ultrasonography and the relatively infrequently requested cytological examination of fine needle aspirated-thyroid gland specimen, were noted if available. Subsequent Clinics' visits were carefully reviewed to assess the thyroid-related medications and the clinical outcome of the patients included in this study. The mean ± SD follow-up period for SAT patients was 7.9 ± 4.7 years.

## 3. Results

**Figure 1:**
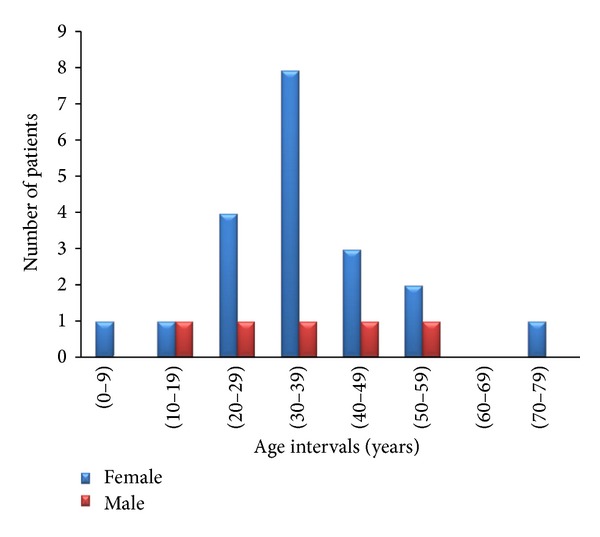
The number of thyroiditis patients in different age intervals.

Reviewing thyroid uptake scan data in KKUH, demonstrated that it was requested for 550 subjects in the current study's specified duration. Among those subjects, low normal, reduced, or absent uptake were reported in 67 subjects (12.2%), and, among the latter, the diagnosis of thyroiditis was recorded in the medical files in 25 patients (37.3%). Thyroiditis patients comprise both genders (20 female and 5 male patients; i.e., 80% of thyroiditis patients are female) with a male to female ratio of 1 : 4 and with their age at presentation ranging from 3 to 78 years old, (average ± SD 35.2 ± 11.2 years). The number of thyroiditis patients in different age intervals is illustrated in Figure 1, and it shows that 75% of patients in the female group were middle age at time of presentation (age range of 20–47 years old). Similarly, 60% of patients in the male group were middle age at time of presentation (age range of 24–44 years old). In both genders 72% of patients were middle age at time of presentation.

When we classified thyroiditis patients based on the presence or absence of neck pain, 8 subjects fulfill the criteria of thyroiditis with thyroid pain and tenderness, while the remaining 17 subjects fulfill the criteria of painless thyroiditis. Among the 8 painful thyroiditis subjects, 7 individuals were SAT—those will be analyzed in more detail below—while one subject (a child) was diagnosed with acute bacterial (suppurative, infectious) thyroiditis. The latter was a 3-year-old girl who presented with severe pain and tenderness in the neck region accompanied by fever and thyroid swelling. Laboratory investigations demonstrated elevated ESR, leukocytosis, and normal TFT. Thyroid investigations demonstrated low thyroid uptake, and both ultrasound and fine needle aspiration and cytological examination have confirmed the presence of a thyroid abscess due to bacterial infection. Other reported categories of painful thyroiditis were not represented in our retrieved medical records, such as traumatic, radiation, or the more rare cases of painful Hashimoto's thyroiditis.

Among those with painless thyroiditis, two subjects were diagnosed as Hashimoto's thyroiditis based on the clinical findings, the laboratory results especially those of high and persistent titer of AMA; one subject was diagnosed as postpartum thyroiditis; one subject had lithium-induced thyroiditis, and 13 subjects fulfilled the criteria of painless sporadic thyroiditis. Both Hashimoto's thyroiditis cases were female in the thirties of their age, one of them suffered as well from other allergic and inflammatory disorders (adult Still's disease, allergic rhinitis, and chronic hepatitis). The laboratory investigations of both cases have demonstrated elevated ESR, anemia, lymphocytosis, and monocytosis. Their TFT results differ, one Hashimoto's thyroiditis patient had overt thyrotoxicosis upon presentation and was actually treated with antithyroid drug (ATD) and corticosteroid, while the other subject had hypothyroidism upon presentation and was started on thyroxine replacement therapy. Both subjects manifested low thyroid uptake and the characteristic ultrasonography and cytological findings of Hashimoto's thyroiditis. Among the painless thyroiditis, one subject—a female 28-year-old at time of presentation—presented with goiter. Upon investigation, she had low thyroid uptake and was diagnosed as painless postpartum thyroiditis. Her condition developed into permanent hypothyroidism which necessitated thyroxin replacement therapy. A case of drug-induced thyroiditis was among the retrieved medical records. This was 30 years old at time of presentation, psychiatric female patient who developed lithium-induced thyroiditis manifested by low thyroid uptake, transient thyrotoxicosis, and a transient AMA autoantibody. The condition was spontaneously resolved, and the AMA titer turned negative in 2 years. The remaining painless thyroiditis subjects were categorized as painless sporadic thyroiditis, who manifested the characteristic clinical, laboratory, thyroid uptake, imaging, and cytological criteria.

Among the thyroiditis subjects in general, the clinical presentation varied, with different combinations as demonstrated in Table 1. The most frequently reported symptoms and signs were palpitation, goiter, and weight change, while those least frequently reported were ear and face pain and shortness of breath.  Table 2 demonstrates the results of laboratory investigations and of the thyroid uptake and scan of the thyroiditis cases as a whole.

In addition to the thyroid uptake scan, results of other investigation as thyroid ultrasonography and fine needle aspiration (FNA) were available for some of the patients. Thyroid ultrasonography was requested for 10 patients out of the 25 patients diagnosed as thyroiditis (40%). Of these, small hypoechoic or anechoic nodules less than 1 cm and irregular thyroid surface were mainly described. Furthermore, FNA and cytological examination was done for 6 patients (24%) showing colloid nodule (2 patients), a colloid nodule with cystic changes (1 patient), acute inflammatory cells and abscess (1 patient), and lymphocytic infiltration with criteria of chronic inflammation (2 patients).

Further reviewing the records of the whole group of thyroiditis patients to document the treatment approach revealed that in the initial thyrotoxic phase, *β*-blockers were prescribed for 8 of the 25 patients (32%). Only one patient (4%) was prescribed gradually tapering dose of corticosteroids. In addition, during the course of the disease, 6 patients were prescribed thyroxine; one of them during the hypothyroid phase of the disease, while in the remaining 5 patients, thyroxine was prescribed for permanent hypothyroidism. On the other hand, ATD (carbimazole) was transiently prescribed for 5 patients and were stopped upon normalization of TFT.

Careful revision for the details of the subsequent patients' clinics visits for a follow-up period of 7 ± 4 years (average ± SD) to record the clinical outcome has revealed that euthyroid state and resolution of thyroiditis was reported in 20 patients (80%), while permanent hypothyroidism occurred in 5 patients (20%).

We further analyzed the painful SAT group records separately to be compared to the results from the western region of the Kingdom.

**Figure 2:**
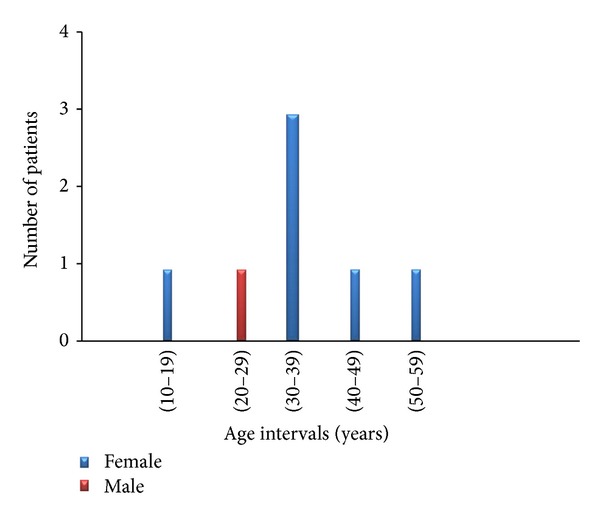
The number of SAT patients in different age intervals.

There is obvious similarity of both gender and age distribution of the SAT patients (Figure 2) to those of the thyroiditis group as a whole (Figure 1); SAT is more represented in females (6 females and 1 male patients; i.e., 85.7% of SAT patients are female). The subjects' age at presentation ranges from 16 to 54 years old, (average ± SD 34.7 ± 8.6 years). The number of SAT patients in different age intervals is illustrated in Figure 2, and it shows that 71.4% of SAT patients in both genders were middle age at time of presentation.

The symptoms and signs of SAT at time of presentation varied, with palpitation and sore throat present in 62.5% of subjects, weight loss in 50%, goiter, anxiety, fatigability and heat intolerance in 37.5%, excessive sweating and hoarseness of voice in 25%; and the least reported clinical presentations were tremors, fever, mood change, insomnia, loss of hair, and ear and face pain (present in only 12.5% of the SAT subjects).

Table 3 demonstrates the results of laboratory investigations and of the thyroid uptake and scan. It shows similar pattern as that of the whole thyroiditis group (Table 2) but with greater elevation of FT4, FT3, and ESR average values and greater reduction of the thyroid uptake average value in the SAT group relative to the whole thyroiditis group. Regarding the treatment modality, it was reported that *β*-blockers were prescribed to 57% and NSAID to 29% of SAT cases in the initial thyrotoxic phase. Corticosteroids were not prescribed to any of the SAT patients. It is also noted that ATD were transiently prescribed for 3 out of the 7 patients of painful SAT in the initial thyrotoxic phase. Thyroxine was also transiently prescribed to 2 SAT patients. Follow-up period of 7.9 ± 4.7 years (average ± SD) demonstrated that 6 out of the 7 SAT patients (85.7%) had recovered, whether spontaneously (4 patients) or after transient treatment with thyroxine (2 patients), while 1 patient had developed permanent hypothyroidism for which she has been prescribed thyroxine replacement therapy.

## 4. Discussion

There is some controversy around the classifying terminology for different forms of thyroiditis. We have adopted the most accepted nomenclature for this group of thyroid disorders. The current work's experimental approach was to retrieve all the medical records in a specified period of time that contain results of the thyroid uptake and scan, which is frequently requested to confirm the clinical and laboratory diagnoses of thyrotoxicosis and to start the treatment accordingly. Therefore, using it in combination with the clinical findings, for example, thyroid pain and tenderness, and laboratory results, for example, ESR and TFT, dominate the major diagnostic and therapeutic considerations in our medical institute as well as elsewhere in the world.

The term painful thyroiditis mainly includes SAT and infectious or suppurative thyroiditis. Other rare conditions include traumatic, radiation, and the unusual painful Hashimoto's thyroiditis. On the other hand, painless—or silent—thyroiditis is sometimes called lymphocytic thyroiditis based on the pathological findings of this condition, and it includes various subcategories as previously mentioned [1].

In the present study, we initially analyzed all thyroid disorders for which thyroid uptake and scan demonstrated low normal, reduced, or absent uptake. Among 25 individuals with this abnormal uptake retrieved in a period of 8 years, 17 subjects (68%) were presented as painless thyroiditis and 8 subjects (32%) were presented as painful thyroiditis. Different pathologic conditions of retrieved painless thyroiditis encompass the following: 13 subjects (52%) with painless sporadic thyroiditis, 2 subjects (8%) with Hashimoto's thyroiditis, 1 subject (4%) with painless postpartum thyroiditis, and 1 subject (4%) with painless drug- (lithium) induced thyroiditis. No Riedel (fibrous) thyroiditis was retrieved among the included medical records of the current study. The single case of lithium-induced thyroiditis transiently developed autoantibodies to the thyroid gland (AMA). This is similar to other reports in the literature, where damage to the thyroid gland due to destructive thyroiditis results in releasing of thyroid follicles contents and triggering the immune system to react [8].

Regarding painful thyroiditis, 7 subjects (28%) were diagnosed as SAT and 1 subject (4%) with infectious (suppurative) thyroiditis. No radiation, traumatic, or painful Hashimoto's thyroiditis were diagnosed among the included records of this work.

We further analyzed the painful SAT in more detail, as this condition was previously studied in a different region of the Kingdom of Saudi Arabia [7], and our aim was to compare both studies' outcomes in an effort to detect geographical or environmental differences, if any. A discussion of this analysis and consequent recommendation is in the following sections.

SAT is a self-limiting inflammatory disorder of the thyroid gland, with a possible viral cause. Relative to other—more common—thyroid disorders, SAT is considered uncommon, with the incidence rate of 3 cases per 100,000 per year as reported in a study performed in Olmsted County, Minnesota, USA [5]. In the Denmark, a prospective population-based study that was recently published focusing on patients with overt hyperthyroidism in 2 cities with slightly different iodine deficiency status has confirmed the previously reported low incidence of SAT. Among the Denmark's cohorts of patients with various nosological types of hyperthyroidism, SAT only represented 2.3% [9]. There is only one published study in the Kingdom of Saudi Arabia that reported on SAT cases in the western region of the Kingdom [7], and none was reported in other districts including the central province where the capital city, Riyadh, is located. In the western region, 23 patients with SAT were retrieved in a duration of 2 years and were followed up for 2 years. Indeed, no wide scale community-based studies have been conducted anywhere in the Kingdom in order to estimate the standardized incidence rate of SAT in the Kingdom of Saudi Arabia. In the present study, the approach adopted allowed us to analyze the clinical presentation and outcome of confirmed cases of SAT that have been diagnosed in KKUH and to compare these data to other investigators' published reports. We retrieved 7 cases of confirmed SAT diagnosis in 8-year duration by revising the medical records of 550 patients who underwent a thyroid uptake scan.

Consistent with several published reports [5–7, 10–13], our findings demonstrate a clear gender preference, where women are more affected than men with similar gender ratio as reported in the literature. Similarly, an age preference was noted in the present study with the age group more affected by SAT being the middle age adults for both females and males. This is also in line with data reported by other groups [5, 6, 9, 12, 13].

In the present study, the patients' leading reported symptoms and signs were those related to thyrotoxicosis; this is consistent with other reports for SAT [12]. One patient presented as a case of Fever of Unknown Origin, an unusual presentation that was previously reported in the literature [14, 15].

It is noted that laboratory measurement of some of parameters assessed by other hospitals or suggested in the literature, for instance circulating thyroglobulin and CRP levels [16, 17], respectively, are not requested in our center. Future studies might be required to assess the potential advantages, if any, and to estimate the cost-effectiveness of requesting such parameters for suspected thyroiditis patients. In line with other reports on SAT, FNA cytology is not routinely requested for the disease diagnosis, and it is used only in cases where the diagnosis of painful/tender neck mass is ambiguous.

As mentioned earlier, a report on painful SAT cases in the western province of the Kingdom was previously published [7]. The Kingdom's western province is located on the Red Sea coast and is therefore environmentally different than the central one, where there are no seas or permanent rivers. Both studies were not designed to be epidemiological ones, and hence no incidence rate per population per year can be obtained from either study. The western province study reported similar age distribution and gender preference trend but different value for the female to male ratio than in the current study (female to male ratio 1.9 : 1 in the western region and 6 : 1 in the central region). The laboratory investigation results in general follow the same pattern in both regions of the Kingdom. On the other hand, long term clinical outcome differs in both regions. In the present study, permanent hypothyroidism was reported in 5 patients of the whole thyroiditis group (20%), and in 1 patient of the painful SAT group. The western region's study reported no permanent hypothyroidism in its painful SAT group. The long follow-up duration for the patients' clinical outcome in our work relative to the study performed in the western province is considered a strong aspect of the present study and might explain the difference observed in the percent of patients who developed permanent hypothyroidism along the years as compared to the western province study. Furthermore, the different therapeutic approach in the initial phase of the painful SAT between both regions might be a partial explanation for different clinical outcome. The study from western province reported that anti-inflammatory drugs were frequently prescribed as follows: 35% of patients were treated with corticosteroids alone, 30% with NSAID only, and 35% with both NSAID and corticosteroids. In addition, almost all their patients were prescribed a *β*-blocker as a symptomatic therapy, and 13% of their patients were treated initially with ATD. On the other hand, the present study demonstrates that in the initial thyrotoxic phase of painful SAT, *β*-blockers were prescribed for 57% of cases and NSAID for 29% of patients. Prescribing corticosteroids does not seem to be a common practice in our center. It is also noted that ATD were transiently prescribed for 3 out of the 7 patients of painful SAT in the initial thyrotoxic phase. In addition, among the painful SAT subjects, thyroxine was prescribed transiently to 2 patients and permanently to a third patient who developed permanent hypothyroidism.

Considering the different location and lifestyle of the central and the western regions of the country, it is intriguing to hypothesize that the clinical outcome differences between the current study and that of Qari and Maimani are possibly partially due to the difference in iodine deficiency between these two regions [18]. In fact, studies on iodine status from different provinces in the Kingdom are scarce and more thorough analysis is needed [19]. However, the small number of available studies has shown a clear geographical difference in iodine state in the Kingdom, as measured by the urinary iodine concentration and the percentage of subjects with a low urinary iodine concentration. Clinically, the provincial difference in the iodine status was associated with a corresponding difference in the prevalence and severity of goiter. In different regions of the world, a similar association was reported. Carlé and coworkers have recently conducted an epidemiological study of subtypes of hyperthyroidism in 2 cities with different iodine deficiency status in Denmark [9]. They have reported a significantly higher standardized incidence rate per 100,000 person-years in the city with more iodine deficiency. Based on the unpredicted nature of SAT and the difference in the clinical outcomes between the western and central provinces of the Kingdom, a nation-wide well-designed epidemiological study would be expected to provide population standardized statistical analysis of thyroid disorders including SAT in different regions of the Kingdom and equally important to help in establishing a practical guidelines for management of this disease and other thyroid disorders.

The present study clearly demonstrates that prescribing corticosteroid for SAT patients is not a common practice in our center even in the early phase of the disease where corticosteroids might be considered as effective symptomatic therapy. In fact, prescribing *β*-blockers is more commonly resorted to aiming at controlling the initial thyrotoxic manifestations of the disease. The management approaches reported in the literature for SAT indicate that some patients require no treatment, while many others frequently require NSAID as symptomatic analgesic and anti-inflammatory agents. If the symptoms persist despite the use of NSAID or in cases with severe symptoms, corticosteroids are usually prescribed only after excluding acute suppurative thyroiditis [20]. It is quite interesting to study the correlation between the therapeutic approach and the clinical outcomes for patients with SAT. This has not been widely studied, except for few reports. Fatourechi and colleagues have reported that among their cohort, prescribing corticosteroids did not protect against the occurrence of transient hypothyroidism in the early phase of the disease. As a matter of fact, they reported that long term hypothyroidism was significantly more common in the group who received corticosteroid therapy [5]. In our group, none of the SAT patients received corticosteroids and thus we cannot comment on the association between corticosteroid and a particular clinical outcome.

The current study has strength points and some limitations. One limitation is imposed by the approach adopted. Since SAT could be diagnosed clinically without performing a thyroid uptake scan, using the latter as the method for retrieving medical records for patients to be included in the study might have underestimated the number of SAT cases diagnosed in our region during the 8 years specified. On the other hand, focusing on confirmed cases and reporting on the clinical presentation and outcome for a long follow-up period are clearly strength points in the current work.

## 5. Conclusions

In conclusion, our retrospective review study shows that SAT is an uncommon disease in the central province of the Kingdom of Saudi Arabia. The disease follows the differential age and gender preferences as reported in the western province of the Kingdom and in different parts of the world. Symptomatic corticosteroid therapy is not a common practice in treating the thyrotoxic initial phase of the disease in the central region as compared to the western region of the Kingdom. On the other hand, permanent hypothyroidism is not uncommon among the SAT patients followed up for several years in the central as compared to the western region of the country. Results obtained from this study highlight the need for a nation-wide well-designed epidemiological study for all thyroid disorders including SAT that would be expected to provide population-standardized statistical information and help in establishing practical guidelines for the management of thyroid disorders in various regions of the country.

## Figures and Tables

**Table 1:** Clinical presentation of patients with thyroiditis.

Clinical presentation (symptoms and signs)	Percent of total thyroiditis subjects
Palpitation (and tachycardia)	50
Goiter and tenderness	45.8
Weight change	41.7
Neck pain, sore throat, and painful deglutition	32
Anxiety, nervousness, and irritability	25
Fatigability	25
Heat intolerance	20.8
Change in appetite	16.7
Tremors	16
Fever	12.5
Excessive sweating	12.5
Mood change (depression)	12.5
Insomnia	12.5
Hoarseness of voice	12.5
Nausea and vomiting	8.3
chest pain	8.3
Loss of hair	8.3
Ear and face pain	4.2
Shortness of breath	4.2

**Table 2:** Laboratory and thyroid uptake scan results at time of presentation for thyroiditis patients.

Laboratory test	Normal value	Mean ± SD	Range
FT4 (pmol/L)	10.3–25.8	37.8 ± 20.5	(13.2–100)
FT3 (pmol/L)	4.6–9.2	17.1 ± 7.9	(6.8–29)
TSH (mIU/L)	0.25–5	0.3 ± 0.4	(0.005–1.83)
ESR (mm/h)	3–9	53.1 ± 37.3	(6.0–144.0)
Thyroid uptake and scan (%)	Up to 2	0.4 ± 0.36	(0.00–1.85)

FT4: free thyroxine; FT3: free triiodothyronine; TSH: thyroid stimulating hormone; ESR: erythrocyte sedimentation rate.

**Table 3:** Laboratory and thyroid uptake scan results at time of presentation for subacute thyroiditis patients.

Laboratory test	Normal value	Mean ± SD	Range
FT4 (pmol/L)	10.3–25.8	53.7 ± 31.4	(14.5–100)
FT3 (pmol/L)	4.6–9.2	20.2 ± 8.9	(6.8–29.0)
TSH (mIU/L)	0.25–5	0.47 ± 0.64	(0.01–1.67)
ESR (mm/h)	3–9	68.7 ± 49.1	(6.0–144.0)
Thyroid uptake and scan (%)	Up to 2	0.09 ± 0.13	(0.0–0.32)

FT4: free thyroxine; FT3: free triiodothyronine; TSH: thyroid stimulating hormone; ESR: erythrocyte sedimentation rate.

## References

[1] Pearce EN, Farwell AP, Braverman LE (2003). Thyroiditis. *The New England Journal of Medicine*.

[2] Yasuji I (2013). Subacute thyroiditis in a patient with juvenile idiopathic arthritis undergoing etanercept treatment: a case report and review of the literature. *Modern Rheumatology*.

[3] Nyulassy S, Hnilica P, Buc M (1977). Subacute (de Quervain’s) thyroiditis: association with HLA Bw35 antigen and abnormalities of the complement system, immunoglobulins and other serum proteins. *Journal of Clinical Endocrinology and Metabolism*.

[4] Samuels MH (2012). Subacute, silent, and postpartum thyroiditis. *Medical Clinics of North America*.

[5] Fatourechi V, Aniszewski JP, Fatourechi GZE, Atkinson EJ, Jacobsen SJ (2003). Clinical features and outcome of subacute thyroiditis in an incidence cohort: Olmsted County, Minnesota, study. *Journal of Clinical Endocrinology and Metabolism*.

[6] Cappelli C, Pirola I, Gandossi E, Formenti A, Agosti B, Castellano M (2013). Ultrasound findings of subacute thyroiditis: a single institution retrospective review. *Acta Radiologica*.

[7] Qari FA, Maimani AA (2005). Subacute thyroiditis in Western Saudi Arabia. *Saudi Medical Journal*.

[8] Iitaka M, Momotani N, Hisaoka T (1998). TSH receptor antibody-associated thyroid dysfunction following subacute thyroiditis. *Clinical Endocrinology*.

[9] Carlé A, Pedersen IB, Knudsen N (2011). Epidemiology of subtypes of hyperthyroidism in Denmark: a population-based study. *European Journal of Endocrinology*.

[10] Benbassat CA, Olchovsky D, Tsvetov G, Shimon I (2007). Subacute thyroiditis: clinical characteristics and treatment outcome in fifty-six consecutive patients diagnosed between 1999 and 2005. *Journal of Endocrinological Investigation*.

[11] Nishihara E, Ohye H, Amino N (2008). Clinical characteristics of 852 patients with subacute thyroiditis before treatment. *Internal Medicine*.

[12] Schenke S, Klett R, Braun S, Zimny M (2013). Thyroiditis de Quervain. Are there predictive factors for long-term hormone-replacement?. *Nuklearmedizin*.

[13] Erdem N, Erdogan M, Ozbek M (2007). Demographic and clinical features of patients with subacute thyroiditis: results of 169 patients from a single University Center in Turkey. *Journal of Endocrinological Investigation*.

[14] Karachalios GN, Amantos K, Kanakis KV, Deliousis A, Karachaliou IG, Zacharof AK (2010). Subacute thyroiditis presenting as fever of unknown origin. *International Journal of Clinical Practice*.

[15] Cunha BA, Chak A, Strollo S (2010). Fever of unknown origin (FUO): De Quervain’s subacute thyroiditis with highly elevated ferritin levels mimicking temporal arteritis (TA). *Heart and Lung*.

[16] Pearce EN, Bogazzi F, Martino E (2003). The prevalence of elevated serum C-reactive protein levels in inflammatory and noninflammatory thyroid disease. *Thyroid*.

[17] Rao NL, Shetty S, Upadhyay K (2010). Salivary C-reactive protein in Hashimoto’s thyroiditis and subacute thyroiditis. *International Journal of Inflammation*.

[18] Al-Nuaim AR, Al-Mazrou Y, Kamel M, Al-Attas O, Al-Daghari N, Sulimani R (1997). Iodine deficiency in Saudi Arabia. *Annals of Saudi Medicine*.

[19] Madani KA, Al-Amoudi NS, Kumosani TA (2000). The state of nutrition in Saudi Arabia. *Nutrition and Health*.

[20] Lazarus J, Hennessey J Acute and Subacute, and Riedel’s Thyroiditis. http://www.thyroidmanager.org/.

